# Pelvic lymph node dissection for cervical or bladder cancer: embedding residual fat tissue offers no added value

**DOI:** 10.1007/s00428-023-03559-w

**Published:** 2023-05-15

**Authors:** Jasmijn Vaneman, Heleen J. van Beekhuizen, Joost L. Boormans, Patricia C. Ewing-Graham, Geert J. L. H. van Leenders, Ramon G. V. Smolders, H (Lena) C. van Doorn

**Affiliations:** 1 Department of Gynecologic Oncology, Erasmus MC Cancer Institute, University Medical Center Rotterdam, Molewaterplein 40, 3015GD Rotterdam, The Netherlands; 2 Department of Urology, Erasmus MC Cancer Institute, University Medical Center, Molewaterplein 40, 3015GD Rotterdam, The Netherlands; 3 Department of Pathology, Erasmus MC Cancer Institute, University Medical Center Rotterdam, Molewaterplein 40, 3015GD Rotterdam, The Netherlands

**Keywords:** Bladder cancer, Cervical cancer, Metastatic lymph nodes, Pathology, Residual fatty tissue, Pelvic node dissection

## Abstract

Diagnosis of lymph node metastases in pelvic lymph node dissection (PLND) is important for staging and treatment. Standard practice is to submit visible or palpable lymph nodes for histology. We assessed the added value of embedding all residual fatty tissue.

Patients (*n* = 85) who underwent PLND for cervical (*n* = 50) or bladder cancer (*n* = 35) between 2017 and 2019 were included. Study approval was obtained (MEC-2022-0156, 18.03.2022, retrospectively registered).

The median lymph node yield with conventional pathological dissection was 21 nodes (Interquartile range (IQR) 18–28). This led to discovery of positive lymph nodes in 17 (20%) patients. Extended pathological assessment found 7 (IQR 3-12) additional nodes, but did not result in identification of more node metastases.

Histopathological analysis of residual fatty tissue harvested at PLND resulted in an increased lymph node yield, but not in the detection of additional lymph node metastases.

## Introduction

Pelvic lymph node dissection (PLND) is performed for cervical and bladder carcinoma. The number of lymph nodes removed during surgery is often seen as an indication of the quality and/or the radicality of the surgery [1, 2]. Not only is the presence of positive lymph nodes important for staging and for further treatment [3], but also the overall lymph node count has been found to correlate with better clinical outcome [4]. There are several factors that may contribute to the number of lymph nodes counted: variation from patient to patient or from one surgeon to another, the surgical technique used, and the approach of the pathologist, including tissue processing technique [2].

Standard pathology practice is to closely examine the submitted lymph node dissection for macroscopically visible or palpable lymph nodes, and embed these for microscopic analysis. The remaining fatty tissue is stored without being submitted for microscopy. As an alternative, the fat can entirely be embedded, processed and examined for the presence of lymph nodes that were not palpable or visible [1]. While this second approach may yield higher numbers of small lymph nodes, it is more time-consuming and costly, and the question is whether the findings of any extra small nodes identified are clinically relevant.

Between June 2017 and June 2019, the pathology department of the Erasmus MC, assessed the residual fatty tissue after dissecting the lymph nodes in PLND specimens submitted with a radical hysterectomy or a radical cystectomy for cervical or bladder cancer. We investigate the effect of totally embedding all tissue after a PLND to answer the question whether such an extensive analysis would reveal more tumor positive nodes.

## Methods

### Study design

This is a retrospective cohort study comprising patients treated with a PLND for cervical or bladder cancer between June 2017 and June 2019. A standard pathology protocol applied. Small nodes were bisected, or enclosed whole without bisecting if very small. Larger nodes were sliced perpendicular to the long axis. How the nodes were handled was described accurately in the macroscopic description of the specimen. All nodes were totally enclosed, and one section was cut per cassette. No levels were cut. We included all patients in whom extended pathology assessment of the nodes was performed. Study approval was obtained at the Erasmus (MEC-2022-0156, 18.03.2022, retrospectively registered).

### Tissue handing

The lymph node dissection specimen was closely examined for macroscopically visible and/or palpable lymph nodes. These nodes were embedded in containers for processing according to the institution’s standard operating procedure (C-PA). In the extended pathology assessment (E-PA), the residual fatty tissue was totally embedded in additional containers.

### Data collection

From the electronic patient files the following data were retrieved. Patient characteristics (age at diagnosis and body mass index (BMI)), pre- and postoperative histological findings, type of surgery, and postoperative TNM stage. For cervical cancer, the FIGO stage was used, and for bladder cancer the WHO classification [5, 6]. Additional containers (E-PA) were counted to estimate the extra costs. The extra costs were calculated by multiplying the number of extra containers by the estimated cost per container (€22.03). A case report form (CRF) was filled in for each patient and double entry of data was performed using an electronic database capture system (CastorEDC, The Netherlands). Any items missing from the patient’s file were recorded as ‘missing’ or ‘unknown’.

## Results

Between June 2017 and June 2019, 151 patients underwent surgery for cervical cancer, of whom 50 with the extended pathology protocol. In the same period, 119 patients underwent surgery for bladder cancer; the extended pathology protocol was used in 35 cases.

### Patient characteristics

Patient characteristics are described in Table 1. In most cases of cervical cancer, the FIGO stage was FIGO IB1 (86.0%). In 19.4% (*n* = 19) lymph vascular space invasion (LVSI) was noted in preoperative biopsy material.

**Table 1:** Baseline patient characteristics of 85 patients with bladder or cervical cancer undergoing radical surgery including pelvic lymph node dissection

	Bladder cancer (*n* = 35)	Cervical cancer (*n* = 50)
Gender, n (%)		
Male	27 (77.1)	
Female	8 (22.9)	50 (100%)
Age, median (IQR)	67 (62–74)	40 (35–46)
BMI, median (IQR)	26.4 (24.0–28.6)	25.2 (22.5–28.2)
Tumor type cervical cancer, n (%)		
Adenocarcinoma		14 (28.0)
Squamous cell carcinoma		31 (62.0)
Adenosquamous carcinoma		2 (4.0)
Neuroendocrine carcinoma		3 (6.0)
Tumor type bladder cancer, n (%)		
Urothelial carcinoma	32 (91.4)	
Squamous cell carcinoma	2 (5.7)	
Neuroendocrine carcinoma	1 (2.9)	
Type of surgery, n (%)^1^		
Open surgery	20 (57.1)	19 (38.0)
Robot assisted surgery	15 (42.9)	31 (62.0)

^1^Radical cystectomy and radical hysterectomy with pelvic node dissection

In the bladder cancer patients, radiography showed at least one enlarged lymph node in 14.3%. The TNM stage varied from T1 to T4, and 40.0% of the patients with bladder cancer received neoadjuvant chemotherapy.

### Postoperative outcomes and lymph node status

Overall, the median number of lymph nodes found by conventional pathological assessment was 21 (IQR 18–28). Complete assessment of all of the residual fatty tissue increased the lymph node yield by 7 (IQR 3–12) (Table 2).

**Table 2:** Postoperative outcome

	(*n* = 85)
Number of lymph nodes conventional PA, median (IQR)	21 (18–28)
Positive lymph nodes conventional PA, n (%)	
Cervical cancer	6 (12.0)
Bladder cancer	11 (31.4)
Number of lymph nodes extended PA, median (IQR)	7 (3–12)
Number of positive lymph nodes extended PA, median (IQR)	0 (0–0)
Total number of lymph nodes, conventional + extended PA, median (IQR)	31 (25–36)
Number of containers extended PA, median (IQR)	12 (8–16)

Lymph nodes were tumor-positive in 17 (20%) patients. All positive lymph nodes were found by conventional pathological assessment. In other words, they were palpable and/or visible. After surgery, the tumor stage was reassigned for six patients with cervical cancer to FIGO stage IIIC (12.0%), when one or more positive lymph nodes were found.

For the extra assessment of the residual fatty tissue, a median of 12 (range 2–33) extra containers were used. In 2020, the pathological department estimated 22.03 euros per container, so that the average cost of extra pathological assessment was 264 euros per patient (range 44–727 euros).

## Discussion

This retrospective, single-center study shows that assessment of the residual fatty tissue does not lead to the finding of more positive lymph nodes, even though the extended pathological assessment resulted in a 29% higher lymph node count. As might be expected, extra lymph nodes discovered in residual fat tended to be very small. It should also be considered that it may be possible in some cases that the ‘extra’ lymph nodes were actually fragments of the palpable lymph nodes that had been left behind after conventional dissection.

The median number of lymph nodes resulting from conventional pathology dissection in our study was 21. This corresponds with what is described in the literature; a mean of 21 to 32 lymph nodes is reported in various studies [7–10].

Both approaches to pathology dissection have their benefits and disadvantages. Using the standard procedure, very small lymph nodes are not visible by macroscopic inspection, resulting in a lower lymph node count [1]. Using the extended approach, smaller lymph nodes are identified, usually resulting in a higher lymph node count, and possibly representing a more accurate reflection of the exact number of lymph nodes present in the specimen. The extended approach is likely to be less influenced by the individual pathologist performing the dissection [11], making it easier to compare different clinics, surgeons, and even different treatment strategies. However, this approach is more time-consuming and costly [1]. The median cost of the extra assessment of the residual fatty tissue was estimated as 264 euros. This may pale into insignificance compared to the cost of the entire treatment, but it is important to be critical where the yield seems minimal.

### Limitations and strengths

This is a retrospective study with several limitations. First, this is a single-center study, and the results may not be generalizable, owing to differences in population and pathological protocols. There was no revision of the pathology; however, the pathology reports are standard and are issued by pathologists experienced in the subspecialty. Although we included 85 patients, our study population may be too small to find an undetected positive node in the residual fatty tissue. We consider it to be a strength that we studied two different tumor types and that two groups of surgeons (gynecologists and urologists) performed the operations. There was no recall bias, outliers were checked and validated. Double entry of data was performed.

### Recommendations

The present study indicates that the current pathology practice (enclosing only those lymph nodes detected by sight and/or palpation) is adequate. There appears to be no clinical relevance to studying residual fatty tissue; no extra positive lymph nodes were found, and there was therefore no effect on further treatment in both cervical and bladder cancer.

## Data Availability

The datasets generated during and/or analysed during the current study are available from the corresponding author on reasonable request.
